# Melatonin-mediated endogenous nitric oxide coordinately boosts stability through proline and nitrogen metabolism, antioxidant capacity, and Na^+^/K^+^ transporters in tomato under NaCl stress

**DOI:** 10.3389/fpls.2023.1135943

**Published:** 2023-03-13

**Authors:** Abazar Ghorbani, Leila Pishkar, Kobra Valed Saravi, Moxian Chen

**Affiliations:** ^1^ National Key Laboratory of Green Pesticide, Key Laboratory of Green Pesticide and Agricultural Bioengineering, Ministry of Education, Center for Research and Development of Fine Chemicals, Guizhou University, Guiyang, China; ^2^ Department of Biology, Islamshahr Branch, Islamic Azad University, Islamshahr, Iran; ^3^ Department of Biology, Damghan branch, Islamic Azad University, Damghan, Iran

**Keywords:** melatonin, nitric oxide, NHX genes, nitrogen metabolism, NaCl stress, *Solanum lycopersicum*

## Abstract

The interactions between nitric oxide (NO) and melatonin in alleviating sodium chloride (NaCl) toxicity in plants are poorly comprehended. Here, the associations between the exogenous application of melatonin and endogenous NO levels in inducing tomato seedlings’ defense response during NaCl toxicity were investigated. The results indicated that the application of melatonin (150 μM) increased height (23.7%) and biomass (32.2%), improved chlorophyll (a (137%) and b (92.8%)), and proline metabolisms, and reduced the contents of superoxide anion radicals (49.6%), hydrogen peroxide (31.4%), malondialdehyde (38%), and electrolyte leakage (32.6%) in 40-day-old tomato seedlings grown under NaCl (150 mM) treatment. Melatonin increased the antioxidant defense system in NaCl-stressed seedlings by increasing the activity of the antioxidant enzymes. Melatonin also improved N metabolism and endogenous NO content in NaCl-stressed seedlings by upregulating the activity of enzymes implicated in N assimilation. Furthermore, melatonin improved ionic balance and reduced Na content in NaCl-exposed seedlings by upregulating the expression of genes involved in K/Na ratio homeostasis (*NHX1-4*) and increasing the accumulation of mineral nutrients (P, N, Ca, and Mg). However, the addition of cPTIO (100 μM; an NO scavenger) reversed the beneficial impacts of melatonin, indicating the effective function of NO in melatonin-induced defense mechanisms in NaCl-stressed tomato seedlings. Therefore, our results revealed that melatonin improves the tolerance of tomato plants during NaCl toxicity by mediating internal NO.

## Graphical Abstract

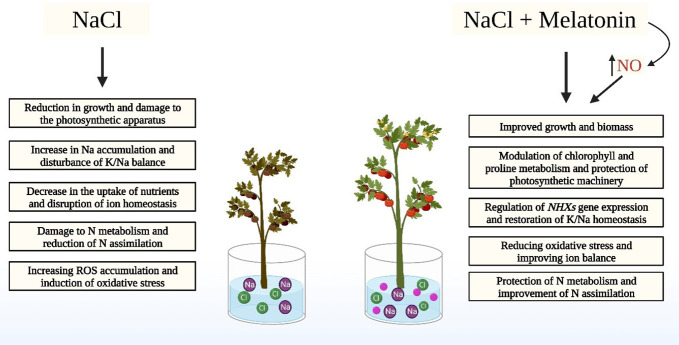

## 1 Introduction

Tomatoes (*Solanum lycopersicum* L.) are an important industrial crop that is susceptible to a variety of environmental stresses. As one of the most important environmental stresses, water or soil salinity is increasingly a serious menace to crop production (Ghorbani et al., 2021; Hao et al., 2021). Unless effective management measures are implemented, roughly 50% of agricultural lands are anticipated to suffer from salt stress by 2050 (Wang et al., 2022). Excess sodium (Na^+^) in irrigation water or soil inhibits photosynthesis, absorption of essential nutrients, and protein synthesis, resulting in serious disruptions in plant vital metabolism, development, and yield (Shabala and Pottosin, 2014). Salt-stressed plants maintain potassium (K^+^) and Na^+^ homeostasis through a variety of mechanisms. Sodium/hydrogen antiporters (NHXs) in the tonoplast regulate the homeostasis of cellular cations, modulate stomatal function, and maintain cellular pH through sequestering Na and adsorbing K into vacuoles (Bassil and Blumwald, 2014). The NHX family includes six members (NHX1-6) in maize and rice and eight members (NHX1-8) in Arabidopsis (Khan et al., 2018). NHX1–4 isoforms were specified in tomato that are implicated in the accumulation of K in vacuoles, with the NHX1 and NHX2 transporters being the most significant (Gálvez et al., 2012). Furthermore, NaCl toxicity can lead to the overproduction of reactive oxygen species (ROS) and damage to various cellular organelles. These destructive effects induced by salinity are reduced by the cellular antioxidant defense system, such as antioxidant enzymes (e.g., peroxidases, catalase, and glutathione reductase), by diminishing the accumulation of free radicals (Li et al., 2017; Ghorbani et al., 2018b).

Melatonin is a plant-synthesized indoleamine that has been discovered to have crucial functions in root and shoot development, seed germination, and circadian growth rhythms (Murch and Erland, 2021). Melatonin also improved plant adaptation to biotic and abiotic stresses such as low temperature (Qari et al., 2022), high temperature (Byeon and Back, 2014), cadmium toxicity (Kaya et al., 2019), and salinity (Ghasemi-Omran et al., 2021). It has been shown that melatonin treatment can maintain ionic homeostasis in apple and maize plants during salinity stress (Li et al., 2012; Jiang et al., 2016). In addition, melatonin has been indicated to be involved in the adjustment of phytohormones and signaling molecules such as gibberellin, hydrogen peroxide (H_2_O_2_), nitric oxide (NO), and abscisic acid in NaCl-exposed plants (Zhang et al., 2021; Liu et al., 2015; Liang et al., 2015). NO has a vital function in plant stress responses as a redox signaling molecule. Melatonin has been demonstrated to modulate plant adaptation to different stresses through interaction with NO metabolism (Fancy et al., 2017; Yan et al., 2020; Feng et al., 2021). Liu et al. (2015) showed that melatonin reduced the levels of H_2_O_2_, free toxic radicals, and malondialdehyde (MDA) and improved the activities of catalase (CAT), ascorbate peroxidase (APX), and superoxide dismutase (SOD) during sodic alkaline toxicity *via* interaction with NO. Yan et al. (2020) suggested that the application of melatonin by inducing nitrate reductase (NR)-synthesized NO up-regulated the H+-pump activity of the vacuole membrane and plasma membrane and, consequently, maintained K^+^/Na^+^ balance in rice under NaCl stress.

Apart from the outstanding defensive effects of NO and melatonin on the induction of plant adaptation under environmental stress, there is no accurate information on the regulatory role of melatonin treatment on NO metabolism in NaCl-stressed tomatoes and/or the function of melatonin and NO interaction in the molecular mechanisms of K/Na hemostasis in tomato plants. Accordingly, we examined the regulation of NO metabolism by melatonin as well as the cross-talk between NO and melatonin in the expression of NHXs transporters and the adjustment of K^+^/Na^+^ balance in tomato plants during NaCl toxicity. In addition, the effects of melatonin and NO interaction on chlorophyll and proline metabolism, the antioxidant machinery, and ionic homeostasis in tomato leaves were investigated under salinity conditions. The findings of this study may provide a new physiological basis for further elucidating the regulatory mechanisms of NaCl toxicity tolerance in tomato plants induced by the melatonin-NO interaction.

## 2 Material and methods

### 2.1 Plant materials and treatments

The surface-sterilized seeds (5% NaClO for 5 min) of tomato (*Solanum lycopersicum* var. Super 2270) were germinated in plastic trays containing autoclaved peat moss (Ghorbani et al., 2022). The 20-day-old tomato seedlings were assigned to hydroponic boxes comprising nutrient solution (1/2-strength Hoagland solution, pH 6.0) (Hoagland and Arnon, 1941). Nutrition solutions (Hoagland solution only and Hoagland solution containing NaCl, melatonin, and cPTIO treatments) were renewed every 3 days. Tomato seedlings were grown in growth chambers with a temperature of 25/22°C (14/10 h) day/night, 275 ± 25 μmol/m^2^/s fluorescent light, and 60% humidity. The following treatments of NaCl (150 mM), melatonin (150 μM), and 2-(4-carboxyphenyl)-4, 4, 5, 5-tetramethylimidazoline-1-oxyl-3-oxide (cPTIO, 100 μM, as an NO scavenger) were added to 27-day-old seedlings (7 days after transfer to hydroponic medium): (i) control; only nutrient solution, (ii) melatonin 150 μM; (iii) melatonin 150 μM + cPTIO 100 μM; (iv) NaCl 150 mM; (v) NaCl 150 mM + melatonin 150 μM; (vi) NaCl 150 mM + cPTIO 100 μM; (vii) NaCl 150 mM + melatonin 150 μM + cPTIO 100 μM. NaCl concentration was obtained from the results of previous research (Ghorbani et al., 2018a), and melatonin and cPTIO concentrations were obtained based on preliminary experiments. Fourteen days after the start of various treatments, the plants were collected and held at -80°C (Ghorbani et al., 2020). Five independent replicates (three replicates for gene expression) were used for sampling for each trait.

### 2.2 Photosynthetic pigments and Fv/Fm index

The procedure of Lichtenthaler (1987) was applied to determine the content of chlorophyll *a*, chlorophyll *b*, and carotenoids using an acetone (80%) solution and readings at 460, 645, and 663 nm. After matching the leaves in the dark for 20 min, the Fv/Fm was detected with a PAM fluorometer (PAM 2500, Walz).

### 2.3 δ-Aminolevulinic acid (ALA) and proline

The leaf content of ALA was specified by estimating the production of porphobilinogen from ALA at 550 nm, as defined by Harel and Klein (1972). The Bates et al. (1973) procedure was employed to specify the contents of proline by sulfosalicylic acid and readings at 520 nm.

### 2.4 Hydrogen peroxide (H_2_O_2_), superoxide anion, and malondialdehyde (MDA)

The procedure of Velikova et al. (2000) was used to appraise the content of H_2_O_2_ using thiobarbituric acid (TCA, 1%) and reading at 390 nm. Superoxide anion radicals were quantified using an extraction solution containing Tris-HCl buffer (50 mM, pH 6.5), nitrobluetetrazolium (0.2 mM), NADH (0.2 mM), and sucrose (250 mM) and readings at 530 nm, as expressed by Achary et al. (2012). After homogenization of fresh tomato leaves in trichloroethanoic acid (10%) and 2-thiobarbituric acid (0.65%) and recording the optical densities at 532 and 600, the leaf content of MDA was estimated following the procedure previously explained by Heath and Packer (1968).

### 2.5 Determination of nitric oxide (NO) content and electrolyte leakage (EL)

The procedure of Zhou et al. (2005) was used to specify the internal level of NO in the leaves using the Griess reagent and the detection of nitrate (NO^2−^) at 540 nm. After preparing the leaf pieces and rinsing them with distilled water, the leaf pieces were placed in tubes containing distilled water on a shaker for 24 hrs. After recording the electrical conductivity (EC1), the tubes were autoclaved at 120°C for 20 min, and then EC2 was recorded. Electrolyte leakage was achieved as per Dionisio-Sese and Tobita (1998): EL (%) = (EC1/EC2) × 100.

### 2.6 Extraction and assay of enzymes

An extraction solution including potassium-phosphate buffer (100 mM, pH 6.8), ethylenediaminetetraacetic acid disodium salt dehydrate (5 mM), N-vinylpyrrolidinone (2%, w/v), and 0.5% TX-100 was operated to homogenize tomato leaf. After centrifugation, the supernatants were utilized to assess the activity of enzymes (Polle et al., 1994).

The leaf activities of SOD, CAT, glutathione reductase (GR), and APX enzymes were obtained by following the procedures previously reported by Foyer and Halliwell (1976); Dhindsa and Matowe (1981); Aebi (1984), and Nakano and Asada (1981).

The leaf activities of nitrite reductase (NiR), nitrate reductase (NR), glutamyl synthetase (GOGAT), and glutamine synthetase (GS) were quantified by Sawhney and Naik (1973); Boland and Benny (1977); Planchet et al. (2005), and Washitani and Sato (1977), respectively.

The procedures formerly described by Jain and Gadre (2004); Costa et al. (2005); Sumithra et al. (2006), and Charest and Phan (1990) were employed to quantify the activities of ALA dehydratase (ALAD), chlorophyllase (Chlase), proline dehydrogenase (ProDH), and Δ1-pyrroline-5-carboxylate synthetase (P5CS) enzymes, respectively.

### 2.7 Chemical analyses

The PFP7 model flame photometry (Jenway, Stone, UK) was operated to measure calcium (Ca^2+^), magnesium (Mg^2+^), and chloride (Cl^−^) ions in leaves and the concentrations of Na^+^ and K^+^ ions in leaves and roots. The phosphomolybdate blue (Murphy and Riley, 1962) and Kjeldahl (1883) methods were used to assess the leaf concentrations of phosphorus (P) and nitrogen (N), respectively.

### 2.8 Gene expression

The Qiagen RNeasy kits were employed to extract total RNA from leaves and roots, following the manufacturer’s instructions. Superscript reverse transcriptase (Invitrogen) and SYBR green PCR master mix (Applied Biosystems) were utilized for cDNA synthesis and qPCR reactions, respectively. The primers for target genes (**Table S1**), *NHX1-4*, and *Actin* (the reference gene) were designed with the Primer3 program. The relative transcript level of the target genes was estimated as per the 2^-ΔΔct^ method with three technical replicates (Livak and Schmittgen, 2001).

### 2.9 Statistical analysis

Data analysis was achieved by SAS 9.1, and the results are displayed as mean ± SD (n = 5; n = 3 for gene expression). The data were computed employing a one-way ANOVA, and the mean differences were specified as per the LSD test (*p* < 0.05).

## 3 Results

### 3.1 Growth and photosynthetic traits

The results displayed that the application of NaCl (150 mM) remarkably decreased the height (29.2%) and the total dry weight (37.1%) compared to control plants. In the absence of NaCl treatment, melatonin (150 μM) treatment did not have a significant effect on the growth and biomass. Regardless, melatonin caused a significant enhancement in height (23.7%) and total dry weight (32.2%) of tomato plants versus plants in the presence of salinity alone. In addition, when cPTIO (100 μM, a NO scavenger) was applied in the presence of melatonin and NaCl, plant height and biomass were decreased compared to their controls (**Table 1**).

**Table 1:** Height, total dry weight, chlorophyll a, chlorophyll b, carotenoids, and Fv/Fm value in tomato seedlings treated with melatonin (150 μM) and cPTIO (100 μM) under NaCl (150 mM) treatment.

	Height (cm)	Total dry weight (g)	Chlorophyll a (mg/gFW)	Chlorophyll b (mg/gFW)	Carotenoids (mg/gFW)	Fv/Fm
CT	20.70 ± 0.87^ab^	3.40 ± 0.18^a^	2.25 ± 0.11^a^	0.933 ± 0.127^a^	0.630 ± 0.046^ab^	0.544 ± 0.014^a^
M	21.83 ± 0.75^a^	3.46 ± 0.13^a^	2.23 ± 0.09^a^	0.950 ± 0.092^a^	0.643 ± 0.050^a^	0.549 ± 0.012^a^
M + cPTIO	20.00 ± 0.82^b^	3.04 ± 0.14^b^	2.12 ± 0.13^a^	0.907 ± 0.084^a^	0.650 ± 0.041^a^	0.524 ± 0.012^b^
S	14.63 ± 0.55^e^	2.14 ± 0.08^d^	0.78 ± 0.09^d^	0.377 ± 0.035^cd^	0.347 ± 0.035^d^	0.403 ± 0.011^e^
S + M	18.10 ± 0.63^c^	2.83 ± 0.12^c^	1.85 ± 0.09^b^	0.727 ± 0.055^b^	0.577 ± 0.035^b^	0.497 ± 0.012^c^
S + cPTIO	13.90 ± 0.40^e^	2.00 ± 0.10^d^	0.64 ± 0.06^d^	0.310 ± 0.020^d^	0.313 ± 0.025^d^	0.395 ± 0.012^e^
S + M + cPTIO	16.10 ± 0.36^d^	2.20 ± 0.07^d^	1.02 ± 0.08^c^	0.453 ± 0.035^c^	0.420 ± 0.031^c^	0.423 ± 0.010^d^

The same letters in each column display no significant differences at the p < 0.05 based on the LSD test (means ± SD, n = 5), CT, control; M, melatonin; S, salinity.

Salinity treatment decreased the levels of chlorophyll *a*, *b*, and carotenoids by 65.3, 59.6, and 45%, respectively, over control plants (**Table 1**). In NaCl-exposed plants, melatonin significantly restored the levels of chlorophyll *a*, *b*, and carotenoids by 137.2, 92.8, and 66.4%, respectively, over salinity treatment alone. Nevertheless, in plants simultaneously treated with salt and melatonin, cPTIO significantly decreased photosynthetic pigments (**Table 1**). NaCl and NaCl+cPTIO treatments significantly reduced Fv/Fm values by 25.9 and 27.4%, respectively, over control plants. Regardless, exogenous use of melatonin improved Fv/Fm values in seedlings exposed to NaCl and NaCl+cPTIO by 23.3 and 7.1%, respectively, over their control treatments (**Table 1**).

### 3.2 Metabolism of chlorophyll and proline

**Figure 1:**
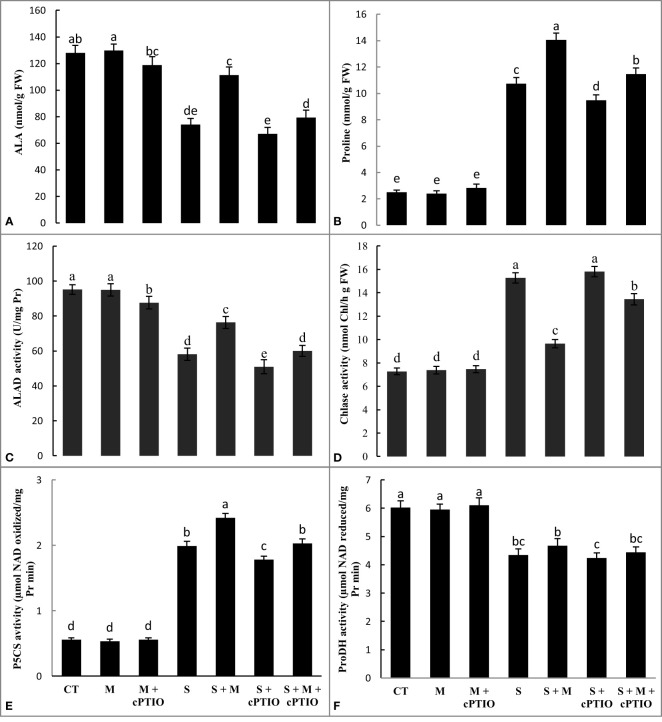
The leaf contents of minolevulinic acid (ALA, **A**) and proline **(B)**, and the leaf activities of aminolevulinic acid dehydratase (ALAD, **C**), chlorophyllase (Chlase, **D**), pyrroline-5-carboxylate synthetase (P5CS, **E**), and proline dehydrogenase (ProDH, **(F)** enzymes in tomato seedlings treated with melatonin (150 μM) and cPTIO (100 μM) under NaCl (150 mM) treatment. Different letters in each column show significant differences at the *p* < 0.05 based on the LSD test (means ± SD, n = 5), CT, control treatment; M, melatonin; S, salinity.

In comparison to the control, NaCl- and NaCl+cPTIO-treated plants had a significant decrease in ALA content. The greatest drop was observed at NaCl+cPTIO treatment. However, melatonin supplementation significantly raised leaf accumulation of ALA in plants treated with salinity and salinity+cPTIO by 50.5 and 18.4%, respectively, compared to their controls (**Figure 1A**). Compared with control plants, the application of NaCl and NaCl+melatonine treatments significantly enhanced the leaf level of proline by 4.2- and 5.6-fold, respectively. Regardless, cPTIO application diminished proline accumulation in the leaves of plants subjected to NaCl and NaCl+melatonine compared to their control (**Figure 1B**).

NaCl and NaCl+cPTIO treatments significantly downregulated the activity of ALAD by 39 and 46.5%, respectively, and upregulated Chlase by 2.1- and 2.2-fold, respectively. However, the exogenous application of melatonin significantly enhanced the activity of ALAD and declined the activity of Chlase in seedlings treated with NaCl+cPTIO and NaCl over their control (**Figures 1C, D**).

The application of NaCl and NaCl+cPTIO treatments increased the activity of P5CS and downregulated the leaf activity of ProDH over control. Regardless, melatonin enhanced the activity of both P5CS and ProDH enzymes over seedlings treated with NaCl+cPTIO and NaCl alone (**Figures 1E, F**).

### 3.3 Antioxidant defense machinery

**Figure 2:**
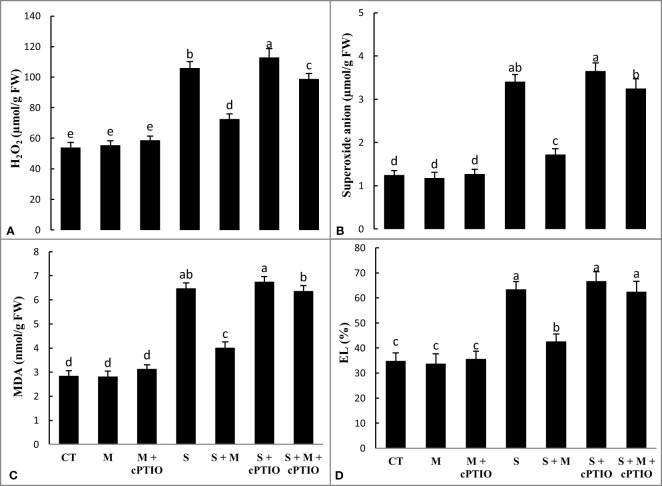
The leaf contents of hydrogen peroxide (H_2_O_2_, **A**), superoxide anion radical **(B)**, malondialdehyde (MDA, **C**), and electrolyte leakage (EL, **D**) in tomato seedlings treated with melatonin (150 μM) and cPTIO (100 μM) under NaCl (150 mM) treatment. Different letters in each column show significant differences at the *p* < 0.05 based on the LSD test (means ± SD, n = 5), CT, control treatment; M, melatonin; S, salinity.

NaCl and NaCl+cPTIO treatments significantly increased the levels of H_2_O_2_ by 96.5 and 109.3% and superoxide anion by 172.8 and 192%, respectively, over control plants. However, melatonin lessened the leaf accumulation of H_2_O_2_ by 31.4 and 12.5% and of peroxide anion by 49.6 and 11% in NaCl and NaCl+cPTIO-stressed plants, respectively, compared to their controls (**Figures 2A, B**). A significant enhancement was observed in the accumulation of MDA at NaCl and NaCl+cPTIO treatments over control, with the highest boost being seen under the NaCl+cPTIO treatment. In both NaCl and NaCl+cPTIO-subjected plants, the supplementation of melatonin significantly lessened MDA accumulation compared to their controls (**Figure 2C**). When tomato seedlings were treated with NaCl and NaCl+cPTIO, electrolyte leakage from leaves increased significantly by 82.2 and 91.8%, respectively, over the control. Melatonin application significantly decreased EL (32.6%) in NaCl-stressed plants but did not induce a significant reduction in NaCl+cPTIO-treated plants (**Figure 2D**).

**Figure 3:**
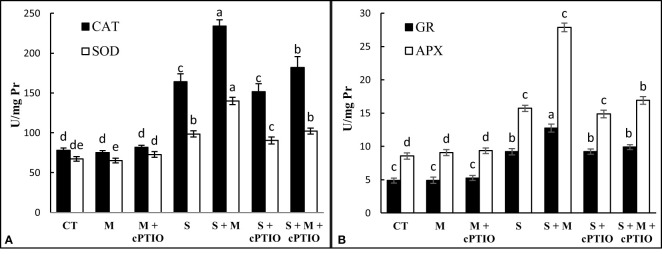
The leaf activities of catalase (CAT, **A**), superoxide dismutase (SOD, **A**), ascorbate peroxidase (APX, **B**), and glutathione reductase (GR, **B**) enzymes in tomato seedlings treated with melatonin (150 μM) and cPTIO (100 μM) under NaCl (150 mM) treatment. Different letters in each column show significant differences at the *p* < 0.05 based on the LSD test (means ± SD, n = 5), CT, control treatment; M, melatonin; S, salinity.

When tomato seedlings were exposed to NaCl and NaCl+cPTIO treatments for 14 days, the activity of CAT, SOD, GR, and APX in the leaves was significantly upregulated over control plants. Regardless, melatonin increased the activity of antioxidant enzymes in both NaCl and NaCl+cPTIO-treated plants compared to their controls (**Figures 3A, B**).

### 3.4 Nitrogen metabolism

**Figure 4:**
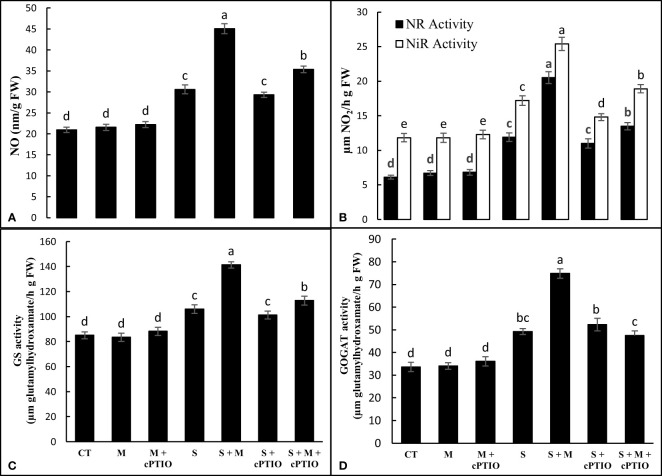
The leaf content of nitric oxide (NO, **A**) and the leaf activities of nitrate reductase (NR, **B**), nitrite reductase (NiR, **B**), glutamine synthetase (GS, **C**), and glutamyl synthetase (GOGAT, **D**) enzymes in tomato seedlings treated with melatonin (150 μM) and cPTIO (100 μM) under NaCl (150 mM) treatment. Different letters in each column show significant differences at the *p* < 0.05 based on the LSD test (means ± SD, n = 5), CT, control treatment; M, melatonin; S, salinity.

When tomato seedlings were subjected to NaCl toxicity, the level of NO was 46.2% higher than that of control seedlings. In NaCl-stressed plants, the exogenous use of melatonin alone and simultaneously with cPTIO enhanced NO content by 47.3 and 15.7%, respectively, compared to NaCl-exposed plants alone. Regardless, cPTIO treatment did not induce a significant effect on NO content (**Figure 4A**). NaCl treatment alone and simultaneously with cPTIO upregulated NR activity by 94.9 and 80.3% and NiR activity by 45.4 and 25.2%, respectively, over control plants. However, melatonin application further enhanced the activity of both NR and NiR enzymes in NaCl- and cPTIO+NaCl-treated plants compared to their control treatments (**Figure 4B**). The activitis of GS and GOGAT displayed significant increases under NaCl and cPTIO+NaCl treatments compared to control, with the highest boost found in NaCl-stressed seedlings. In both NaCl- and cPTIO+NaCl-treated plants, melatonin application significantly enhanced the leaf activity of GS and GOGAT enzymes over their control ones (**Figures 4C, D**).

### 3.5 Mineral nutrients

NaCl treatment alone and simultaneously with cPTIO significantly declined the leaf concentrations of P by 51.3 and 54.4%, N by 39.7 and 43.2%, Ca by 37.7 and 39.7%, and Mg by 48.6 and 50.5%, respectively, over their control. The addition of melatonin caused a further boost in the leaf concentrations of P, N, Ca, and Mg in both NaCl- and cPTIO+NaCl-treated plants (**Table 2**). A 100 and 40% enhancement in the leaf level of Cl was observed in NaCl- and NaCl+melatonine-treated plants, respectively, over control. However, cPTIO application caused a further enhancement in the leaf concentration of Cl in both NaCl- and NaCl+melatonin-treated seedlings over their control (**Table 2**).

**Table 2:** The leaf concentration of mineral nutrients in tomato seedlings treated with melatonin (150 μM) and cPTIO (100 μM) under NaCl (150 mM) treatment.

	P	N	Ca	Mg	Cl
	mg/gDW				
CT	1.95 ± 0.09a	13.92 ± 0.31a	10.45 ± 0.24a	6.79 ± 0.24a	7.46 ± 0.22e
M	1.94 ± 0.12a	14.02 ± 0.27a	10.49 ± 0.27a	6.74 ± 0.24a	7.38 ± 0.22e
M + cPTIO	1.89 ± 0.10a	13.84 ± 0.27a	10.55 ± 0.28a	6.86 ± 0.31a	7.43 ± 0.19e
S	0.95 ± 0.14c	8.39 ± 0.26c	6.51 ± 0.25cd	3.49 ± 0.25cd	14.93 ± 0.31b
S + M	1.55 ± 0.11b	10.53 ± 0.27b	9.44 ± 0.31b	5.49 ± 0.30b	10.45 ± 0.19d
S + cPTIO	0.89 ± 0.12c	7.91 ± 0.18d	6.30 ± 0.27d	3.36 ± 0.23d	15.74 ± 0.24a
S + M + cPTIO	1.01 ± 0.13c	8.84 ± 0.28c	6.93 ± 0.31c	3.93 ± 0.20c	14.08 ± 0.28c

The same letters in each column display no significant differences at the p < 0.05 based on the LSD test (means ± SD, n = 5), CT, control treatment; M, melatonin; S, salinity.

### 3.6 Expression of NHX genes

**Figure 5:**
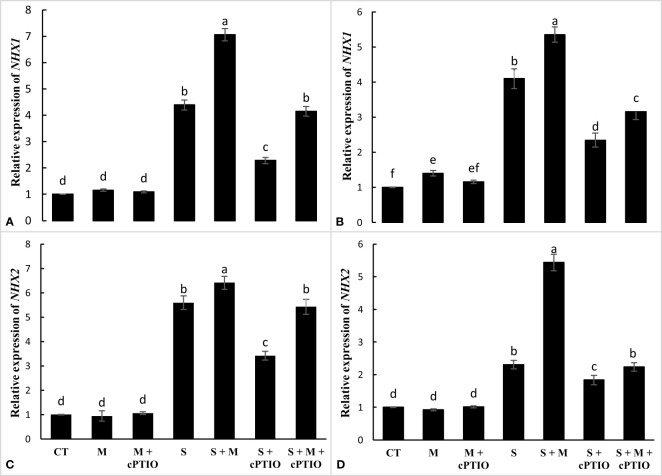
The relative expression of *NHX1* and *NHX2* genes in the root **(A, C)** and leaf **(B, D)** of tomato seedlings treated with melatonin (150 μM) and cPTIO (100 μM) under NaCl (150 mM) treatment. Different letters in each column show significant differences at the *p* < 0.05 based on the LSD test (means ± SD, n = 3), CT, control treatment; M, melatonin; S, salinity.

The results showed that NaCl and NaCl+cPTIO treatments significantly enhanced the relative expression of the *NHX1* gene in the leaves and roots of tomato seedlings, with the highest level of *NHX1* transcription found in NaCl-stressed seedlings. In both NaCl and NaCl+cPTIO treatments, melatonin application significantly enhanced *NHX1* mRNA levels in root and leaf tissues compared to their control treatments (**Figures 5A, B**). NaCl and NaCl+melatonin treatments enhanced the mRNA level of the *NHX2* gene in roots by 5.6- and 6.4-fold and in leaves by 2.3- and 5.4-fold, respectively, over control. In both root and leaf tissues, cPTIO significantly downregulated the expression level of *NHX2* in plants treated with NaCl and NaCl+melatonin (**Figures 5C, D**).

**Figure 6:**
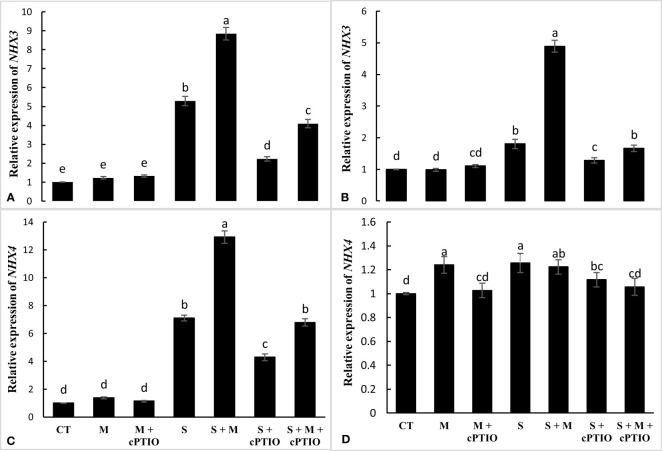
The relative expression of *NHX3* and *NHX4* genes in the root **(A, C)** and leaf **(B, D)** of tomato seedlings treated with melatonin (150 μM) and cPTIO (100 μM) under NaCl (150 mM) treatment. Different letters in each column show significant differences at the *p* < 0.05 based on the LSD test (means ± SD, n = 3), CT, control treatment; M, melatonin; S, salinity.

The relative expression of the *NHX3* gene was significantly enhanced in the roots and leaves of seedlings exposed to NaCl and NaCl-melatonin over control. The highest upregulation was recorded during NaCl+melatonin treatment. In root and leaf, cPTIO application significantly increased the mRNA level of *NHX3* in NaCl and NaCl+melatonin treatments compared to their control (**Figures 6A, B**). A significant upregulation in the relative expression of the *NHX4* gene was found in the roots and leaves of tomato seedlings under NaCl treatments alone and simultaneously with cPTIO over the control. In the roots, melatonin application significantly increased the relative expression of *NHX4* in plants subjected to NaCl and NaCl+cPTIO over controls. Melatonin, on the other hand, had no effect on the mRNA level of *NHX4* in NaCl- and NaCl+cPTIO treated plants (**Figures 6C, D**).

## 4 Discussion

The results showed that NaCl treatment lessened the height and biomass of tomato seedlings, which is in line with earlier declared results on tomato (Ghorbani et al., 2018a), rice (Yan et al., 2020), and cotton (Zhang et al., 2021). NaCl stress has been shown to disrupt various metabolic pathways in plants by interfering with water status and nutrient uptake (Abdelaziza et al., 2019; Ghorbani et al., 2019). Interestingly, melatonin-induced mitigation of NaCl stress was evident through endogenous NO content, verified by the use of cPTIO (a NO scavenger), indicating an inhibition of melatonin-induced impacts in the presence of NaCl toxicity. The beneficial impacts of the exogenous application of melatonin on improving the growth and biomass of various plants have already been documented (Li et al., 2017; Chen et al., 2018; Alharbi et al., 2021). Our results confirm evidence of an interaction between melatonin and NO signaling in diminishing NaCl toxicity in tomato seedlings.

The application of melatonin induced a positive impact on the metabolism of photosynthetic pigments during NaCl toxicity, as evidenced by an increase in ALA accumulation associated with a subsequent upregulation in ALAD activity. The ALAD enzyme is required for the formation of pyrrole compounds by combining two ALA molecules, which is a crucial precursor for chlorophyll production (Killiny et al., 2018). It has been shown that high levels of ALA under stressful conditions can have positive impacts on biomass, the antioxidant defense system, and osmotic regulation under environmental stress (Wu et al., 2019). Moreover, melatonin diminished the chlorophyll degradation process in NaCl-stressed plants by downregulating Chlase activity, which may have protective effects on the performance of the photosynthetic apparatus during NaCl stress. Decreased expression of the *Chlase* gene by melatonin has been earlier documented by Weeda et al. (2014). The positive effect of melatonin on chlorophyll metabolism in NaCl-stressed plants can be caused by reducing oxidative stress and improving the activity of enzymes involved in chlorophyll metabolism (Liu et al., 2020), as well as improving ionic homeostasis and providing more Mg and Fe elements (Yan et al., 2020). However, cPTIO reversed the beneficial impacts of melatonin on chlorophyll metabolism, indicating the regulatory role of melatonin-mediated NO in modulating chlorophyll metabolism during NaCl stress. Additional examinations are needed to specify the exact role of melatonin and its interaction with NO in the biosynthesis of intermediates and chlorophyll metabolism.

It has been shown that the accumulation of osmoregulatory compounds such as proline under environmental stresses maintains ROS scavenging, cellular osmolality, redox homeostasis, and the function of bio-macromoleculars in plants, and is a significant help in improving plant adaptation in stressful conditions (Ghorbani et al., 2018a). NaCl toxicity enhanced the leaf content of proline by decreasing ProDH activity and enhancing P5CS activity, which can be described by putative water stress caused by salinity, as confirmed by Jiang et al. (2021) and Ghorbani et al. (2018a). Guan et al. (2020) revealed that upregulating the expression and activity of enzymes involved in proline biosynthesis (P5CS) increases proline accumulation and, as a result, improves plant adaptation to NaCl toxicity. Melatonin, by modulating enzymes in proline metabolism, provoked a further enhancement in proline in tomato leaves under NaCl stress, which can play a key function in enhancing plant adaptation. Similarly, Jiang et al. (2021) showed that melatonin, by increasing the proline content, protected the photosynthetic apparatus and improved the biomass of the cotton plant during salinity. As a result, employing cPTIO, melatonin-mediated proline metabolism was reversed, confirming the important regulatory function of melatonin-induced NO in the regulation of proline metabolism.

One of the serious damages induced by salinity stress is the excessive production of ROS (H_2_O_2_ and superoxide anion) in plants, which causes serious oxidative damage to important cell components, including biomembranes and the induction of EL. Plants have machinery to balance the endogenous level of toxic free radicals. The NaCl treatment increased the accumulation of toxic compounds such as superoxide anions and H_2_O_2_, resulting in increased MDA and EL, which indicate the induction of oxidative stress in tomato seedlings. NaCl toxicity-induced oxidative stress in tomato (Yin et al., 2019) and stevia (Ghasemi-Omran et al., 2021) plants has also been previously documented. Salinity stress disrupts the function of the photosynthetic apparatus and induces an imbalance between the production and consumption of electrons, causing the transmission of excess electrons to the oxygen molecule and the generation of ROS (AbdElgawad et al., 2016). However, melatonin effectively increased the activity of antioxidant enzymes, thereby reducing the level of ROS and protecting the biomembranes. The helpful impacts of melatonin on the antioxidant machinery and the decline of oxidative stress under salinity stress have already been confirmed by Li et al. (2017) and Jiang et al. (2021). Sun et al. (2021) indicated that melatonin upregulated the expression of antioxidant genes, such as SOD, CAT, APX, and GR, by enhancing the internal contents of NO and, as a result, reduced oxidative stress and enhanced plant biomass under salinity, which is in accordance with the findings of this research. Arora and Bhatla (2017) indicated that melatonin-mediated antioxidant defense in sunflower plants during salinity was dependent on NO. The addition of cPTIO prevented the beneficial impacts of melatonin on the antioxidant machinery, exhibiting the critical function of melatonin-caused internal NO in augmenting the defense system of NaCl-stressed tomatoes.

Key enzymes involved in N and NO metabolism (GOGAT), GS, NiR, and NR) play a crucial function in plant adaptation during environmental stresses (Chen et al., 2021). Melatonin significantly increased the leaf activity of enzymes in N assimilation and the leaf content of NO in NaCl-exposed seedlings, which can effectively improve the tolerance of tomato seedlings. Similar results have already been documented by Talaat (2021) and Ma et al. (2021). An increase in melatonin-induced N metabolism can improve N uptake and transfer between source and sink parts, thereby accelerating the transfer of nutrients between different plant organs under salinity stress (Talaat, 2021). Melatonin was shown to upregulate the expression of *NiR*, *NR*, *GS*, and *GOGAT* genes, which significantly enhanced the adaptation of the plant during stress (Liang et al., 2018). Due to the high sensitivity of GS and GOGAT enzymes to oxidative stress, increasing their activity under melatonin treatment can be due to the melatonin-caused alleviation of oxidative stress (Bose and Howlader, 2020; Zhao et al., 2021). However, cPTIO reduced the activity of enzymes in N assimilation in melatonin-treated plants, indicating the induction of melatonin-mediated N metabolism through the internal NO content under salinity.

Depolarization of the plasma membrane resulting from the influx of Na ions into the cell causes a continuous efflux of K from the cell under salinity stress, which results in a drop in the ratio of K/Na and, consequently, serious damage to vital cell processes (Shabala et al., 2006; Munns and Tester, 2008). NaCl treatment enhanced the accumulation of Na and declined the accumulation of K in the leaf and root of tomato seedlings, which was associated with a decrease in the K/Na ratio. Similar results of reducing the K/Na ratio in tomato (Ghorbani et al., 2019) and rice (Yan et al., 2020) have already been shown. Melatonin effectively reduced Na uptake and transport to the leaves, which maintained K homeostasis and increased the K/Na ratio under NaCl stress. Yan et al. (2020) in rice and Jiang et al. (2016) in maize plants under salt stress reported similar effects of melatonin’s regulatory role in diminishing Na uptake and improving the ratio of K/Na. Melatonin also diminished the translocation of Na to the leaves under salinity, which could be due to decreased Na loading in the xylem (Yan et al., 2021). A report showed that melatonin maintained K homeostasis and improved plant tolerance under salinity by alleviating oxidative stress and regulating K transporters, including HAK5 (Liu et al., 2020). NaCl treatment also reduced the concentration of nutrient minerals (P, N, Mg, and Ca) in the leaves, which could be due to oxidative stress-induced damage to biomembranes, as well as disruption of membrane potential due to the entry of excessive Na levels into the cellular cytoplasm (Ghorbani et al., 2019; Yan et al., 2021). Melatonin significantly improved the concentration of mineral nutrients in NaCl-stressed seedlings, which could result in the reduction of toxic radicals and the stabilization of cell membranes (Liu et al., 2020). The helpful impacts of melatonin on maintaining ionic homeostasis under salinity have been earlier confirmed by Zahedi et al. (2021) and Alharbi et al. (2021). Thus, melatonin improves plant growth and biomass under salinity stress by maintaining ionic homeostasis and reducing Na uptake. Numerous studies have shown that NO treatment effectively reduces Na uptake, improves K/Na, and increases the uptake of mineral nutrients in plants under salinity stress (Zhang et al., 2006; Wang et al., 2009; Hasanuzzaman et al., 2021). The inductive impacts of melatonin on the maintenance of ionic balance and the reduction of Na uptake in cPTIO-treated plants were reversed, indicating that the impacts of melatonin improvement on the maintenance of ionic homeostasis are achieved through NR-mediated NO production.

Salinity stress increases the entry of Na into the cell cytoplasm, which, by damaging cytosolic enzymes, causes a serious disruption in the vital processes of the plant (Fukuda et al., 2011). Therefore, regulating the cytosolic concentrations of Na and K and maintaining the cytosolic ratio of K/Na under salinity can play a critical function in enhancing plant adaptation to salt stress. The three main mechanisms for preventing the cytosolic accumulation of Na include: inhibiting Na influx into the cytoplasm, inducing Na efflux, and sequestering Na in vacuoles (Padan et al., 2001). The *NHX* gene family, located on the tonoplast membrane, is implicated in the compartmentalization of Na into the vacuoles, which can play an important function in reducing the potential toxicity of Na during salinity (Padan et al., 2001; Fukuda et al., 2011). Increased expression of *NHX1*, *NHX2*, *NHX3*, and *NHX4* genes has been shown to decline Na toxicity and, consequently, enhance plant growth under NaCl toxicity (Abdelaziza et al., 2019; Ghorbani et al., 2019). Tan et al. (2021) indicated that the heterologous expression of the melatonin-synthesizing gene was increased by enhancing the expression of *NHXs* and inducing an efflux of Na in the plants under NaCl toxicity, which was accompanied by increased growth and adaptation of the plant under salinity. The results showed that melatonin significantly increased the relative expression of *NHX1*, *NHX2*, *NHX3*, and *NHX4* genes in the root and leaf of tomato seedlings during NaCl toxicity, which could play an important function in Na detoxification by sequestering in vacuoles. Increased expression of *NHX* genes in the root was stronger than in the leaf, which is consistent with a decrease in Na translocation to the leaves. However, when the melatonin-subjected plants were treated with cPTIO, the inducible effects of melatonin on the expression of the *NHX* gene were inhibited, indicating that melatonin-induced upregulation of the *NHX* gene arises through NR-mediated NO during NaCl stress.

## 5 Conclusion

Our findings provide crucial insights into the function of exogenous melatonin and melatonin-mediated internal NO signaling in handling NaCl adaptation in tomato. The results confirmed that exogenous application of melatonin by interaction with endogenous NO increased plant growth and biomass during NaCl stress, indicating that NO as a signaling molecule may be involved downstream of the melatonin-mediated defense response in NaCl-exposed tomato seedlings. To confirm the function of NO downstream of the melatonin-induced signaling pathway, cPTIO was used as a NO scavenger, which showed that cPTIO prevented the beneficial role of melatonin in chlorophyll and proline metabolism, the antioxidant defense system, ionic homeostasis, and modulation of *NHX* gene expression. Therefore, by adjusting N metabolism and enhancing the internal content of NO, melatonin improves chlorophyll and proline metabolism, strengthens the antioxidant defense system, preserves ion balance, and modulates the expression of transporter genes involved in K/Na homeostasis, thus improving the growth and tolerance of tomato seedlings under NaCl toxicity. In the future, the interaction of other enzymes or signaling molecules in increasing the tolerance of abiotic stresses induced by melatonin should be examined.

## Data availability statement

The original contributions presented in the study are included in the article/**Supplementary Material**. Further inquiries can be directed to the corresponding authors.

## Author contributions

Methodology and Conceptualization, AG; Investigation and Validation, AG and LP; Resources, KVS; Analysis, AG; Writing original, AG; Review and Editing, M-XC. All authors contributed to the article and approved the submitted version.
