# Supplementary material for: Melatonin-mediated endogenous nitric oxide coordinately boosts stability through proline and nitrogen metabolism, antioxidant capacity, and Na+/K+ transporters in tomato under NaCl stress
Source: Front Plant Sci. 2023 Mar 13;14:1135943. doi: 10.3389/fpls.2023.1135943 (PMC10040658; doi:10.3389/fpls.2023.1135943)
Supplement: Supplementary file 1 [file Table_1.docx]

**Table S1.** the primers sequences used in qPCR reactions

| Gene name | 5′-primer-3′ | Accession No. |
| --- | --- | --- |
| *NHX1* | F: TGCGGAGATTTTCATTTTCC  R: TGTCATGCTCAGATCGCTTC | AJ306630 |
| *NHX2* | F: GGGCTGCTAATGTGTTTGGT  R: ATTGCCTGACCATGTCCTTC | AJ306631 |
| *NHX3* | F: ACCAAGCACGCTTTTGCTAC  R: AATTGTGGGCTGTCGCTTAC | AM261866 |
| *NHX4* | F: TGTCTGGCTTCTCTGTTTC  R: TCATTAACCCAGCGGTTCTC | AM261867 |
| *Actin* | F: GCCTTAGACTTTGAACAAGAACTCG  R: GGGAAGCTCATAGCTCTTCTCAAC | XM_004239742 |
